# Chronic Recurrent Multifocal Osteomyelitis Causing an Acute Scoliosis

**DOI:** 10.1155/2013/649097

**Published:** 2013-07-31

**Authors:** Alexander Armstrong, Neil Upadhyay, Edward Saxby, Damian Pryce, Nick Steele

**Affiliations:** ^1^Department of Spinal Surgery, Musgrove Park Hospital, Taunton, Somerset TA1 5DA, UK; ^2^Department of Dermatology, Musgrove Park Hospital, Taunton, Somerset TA1 5DA, UK

## Abstract

*Study Design*. A Case Report. *Objective*. We present a 15-year-old girl with an acute atypical scoliosis secondary to chronic recurrent multifocal osteomyelitis (CRMO). *Summary of Background Data*. CRMO is a rare nonpyogenic inflammatory bone condition with unclear aetiology. CRMO mainly affects the metaphyses of long bones, the pelvis, shoulder girdle, and less commonly the spine. *Methods*. Our case presented with a 6-month history of worsening thoracic back pain, asymmetry of her shoulders and abnormal posture. Whole spine radiographs revealed a right atypical thoracic scoliosis. Magnetic Resonance Imaging showed abnormal signal on the short TI inversion recovery (STIR) sequences in multiple vertebrae. A bone biopsy demonstrated evidence of fibrosis and chronic inflammatory changes. Interval MRI scans revealed new oedematous lesions and disappearance of old lesions. Symptoms improved. *Results*. It is important to consider CRMO as an acute cause of atypical scoliosis. Malignancy, pyogenic infections and atypical presentations of juvenile arthritis need excluding. *Conclusion*. This 24-month follow-up describes a rare cause of an atypical scoliosis and fortifies the small amount of the currently available literature. The case highlights the relapsing and remitting nature of CRMO with new lesions developing and older lesions burning out. We advise close radiological surveillance and symptomatic management.

## 1. Introduction

Chronic recurrent multifocal osteomyelitis (CRMO) is a rare nonpyogenic inflammatory bone condition with unclear aetiology. It was first described by Giedion et al. in 1972 as a subacute chronic symmetrical osteomyelitis [1].

CRMO mainly affects the metaphyses of long bones, along with the pelvis, shoulder girdle, and less commonly the spine [2].

CRMO represents 2%–5% of all osteomyelitis cases, mainly affecting young girls with female-to-male ratio of 5 : 1. A 5-year follow-up study revealed the mean age of onset to be 10, with a range of 4–14 years [3]. The natural history of the disease is unpredictable with varying severities and time courses, oscillating between acute exacerbations and spontaneous remission [1, 4].

Other organs are often involved, namely the skin and gastrointestinal tract. Cutaneous manifestations include synovitis, acne, pustulosis, hyperkeratosis, and osteitis (SAPHO) syndrome [5, 6]. In a letter in 1990, Kahn first reported the association of CRMO with inflammatory bowel disease (IBD) reporting that, of the 30 CRMO cases, 4 cases also had Crohn's disease and 1 case had ulcerative colitis [7]. 

CRMO is also associated with other rare autoimmune disease processes. Acute febrile neutrophilic dermatosis (Sweet's syndrome) is characterised by painful erythematous plaques over the face, trunk, and limbs and has been linked with CRMO. Majeed's syndrome is a very rare genetically linked disease, which presents with congenital dyserythropoietic anaemia, Sweet's syndrome, and CRMO seen in a small case series involving a Middle Eastern family [8]. 

We present a 15-year-old girl with an acute scoliosis secondary to CRMO. There appear to be only 2 previously documented similar cases. Moreover, this case has additional features of pustular psoriasis and anaemia, which have required further specialist management. 

**Figure 1:**
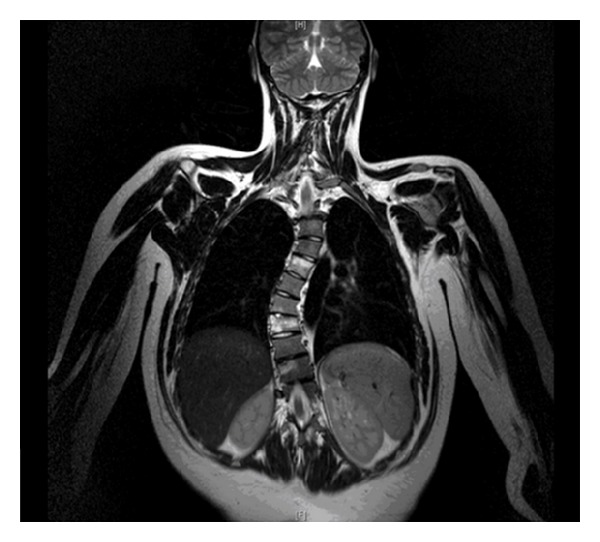
Coronal Thoracic Spine MRI image, T2W. Abnormal high signal in T5 and T8 vertebral bodies with right atypical thoracic scoliosis.

The case is illustrated with a coronal MRI slice, which demonstrates the foci of inflammation in the spine, with a very pronounced thoracic scoliosis (Figure 1).

## 2. Case Presentation

TM is a 15-year-old girl who presented with a 6-month history of steadily deteriorating intermittent back pain mainly affecting her cervicothoracic spine. Her parents had also noticed an asymmetry in her shoulders with abnormal posture. TM was born at term and developed within normal limits. Menarche was at the age 13. She was fully vaccinated and had no previous childhood illnesses. There was no history of trauma.

TM also complained of a recurrent itchy rash affecting her palms and soles over the past 2 years. TM has a positive family history of psoriasis.

On examination, TM stood tall for her age at 167.5 cm. Her right scapula was slightly raised. There was a 10-degree midthoracic rib hump. Upper and lower limb neurology was entirely normal. Respiratory, cardiovascular, and abdominal examinations were normal.

Standing spinal X-rays showed a 36-degree T5–10 right atypical thoracic scoliosis with a 23-degree T10-L3 and T1–T4 compensatory curve. There were no obvious bony deformities.

At this stage, it was planned to follow up TM after a spine MRI and to refer to physios for truncal support exercises.

Blood tests showed a mild normocytic anaemia, and HLA B27 was negative. Spine MRI showed abnormal signal on the STIR sequences in C7, T5, and T8 vertebrae and the right sacral ala. At this point, differential diagnoses were Langerhan's cells histiocytosis, insufficiency fractures, chronic multifocal osteomyelitis, and a leukaemic bone malignancy.

The case was referred for a paediatric oncology opinion and to the Bone Tumour Unit in Birmingham for discussion at the MDT. Both recommended a CT-guided bone biopsy.

TM was admitted for biopsy of her right greater trochanter and skin lesions. The bone biopsy showed evidence of fibrosis and chronic inflammatory changes with no specific diagnosis. There was no evidence of Langerhan's cell histiocytosis or suppurative infection. A punch biopsy of the skin lesions showed pustular psoriasis. All blood cultures were negative.

Under the guidance of the Birmingham Spinal Centre, TM's symptoms were managed conservatively with simple analgesia and truncal physiotherapy. Her symptoms appeared to be improving, and she was clinically examined in the outpatient department at 3 monthly intervals. Whole spine radiographs were taken at 6 monthly intervals to monitor severity of scoliosis, and the MRI was repeated annually to monitor the disease process.

Follow-up whole spine MRIs at 6 months showed some new oedematous lesions around L2 spinous process and sternum. There was also a marked increase in oedema in the lesions previously seen around the right sacroiliac (SI) joint, C7, T5, T7, and T8. These radiological findings correlated with an acute deterioration whilst the patient was on holiday with no clear precipitant. The patient required a wheelchair at one stage due to the pain in her legs.

The interval MRIs at 18 months showed similar signal intensity and size for many of the lesions previously noted in C7, T1, T2, T4-5, and T7-8. There was some improvement in the L2 lesion. New lesions were identified in the left clavicle and multiple ribs bilaterally. Fortunately, symptoms had improved considerably, now tolerating moderate exercise, and the limp had entirely resolved. TM described some mild pain in the lower back and sternum after long periods of sitting.

Her psoriasis improved with the local PUVA treatment managed by a dermatologist. There appeared to be a strong correlation between the improvement in the musculoskeletal and dermatological symptoms.

## 3. Discussion

It is important to consider CRMO as a differential diagnosis when presenting with an acute atypical scoliosis. MRI typically shows multifocal increases in signal on the STIR sequences. High signals can present concurrently or sequentially; therefore, interval scanning is essential to monitor progression of the disease [9]. Malignancy, pyogenic infections, and atypical presentations of juvenile arthritis need to be ruled out, correlating the history, clinical findings and biopsy results. The use of allied professionals in specialist centres is key in formulating this diagnosis. 

CRMO's association with other immune-modulated diseases such as inflammatory bowel disease and psoriasis indicates a likely autoimmune process [10]. Unlike bacterial osteomyelitis, CRMO is not treated with antimicrobials, and anti-inflammatories are shown to improve symptoms. Azithromycin has been used for its anti-inflammatory properties. Indomethacin is an inhibitor of ossification, and a study has shown it to be effective in the treatment of CRMO, with clinical and radiological improvement [11]. The use of systemic steroids during flares and low-dose courses to prevent relapse has been shown to be effective [8].

The natural history of CRMO is poorly understood and may have multiple organ features that require specialist management. This paper fortifies the small amount of the currently available literature on a difficult multifaceted disease to manage.

This 24-month clinical and radiological followup of scoliosis caused by CRMO appears to be the longest in the literature. The case highlights the relapsing and remitting nature of CRMO with new lesions developing and older lesions burning out. Clinically, our patient is improving with less pain, increased exercise tolerance, and no systemic upset. Radiologically, the scoliosis appears to be stable. We advise close radiological surveillance and symptomatic control as the mainstay of management.
